# Towards an integrated and interoperable platform for telehealth and telecare

**Published:** 2012-06-16

**Authors:** Malcolm Clarke, Russell Jones, Joanna Fursse

**Affiliations:** Brunel University, UK; Chorleywood Health Centre, Brunel University, UK; Chorleywood Health Centre, UK

**Keywords:** telehealth, telecare, standards

## Abstract

We present experience of implementing and evaluating a platform purpose designed to integrate interoperable telehealth and telecare. We chose the IEEE 11073 standards for all devices and used ZigBee wireless to support many devices concurrently and exploit its mesh networking to extend range around the entire house. We designed the home gateway to be unobtrusive; in project Hydra we used the smart meter and in other projects (Reaction, inCasa) we have developed a purpose designed plugtop ZigBee to GPRS gateway. All use common protocols and are interoperable.

Technically the projects have been a success, and we have already implemented a wide range of devices on the common platform (BP, weight, SpO_2_, glucose, PIR, medication monitor, bed/chair sensor). Immediate feedback from participants has confirmed our goal of simplicity and convenience of use (and thus encourage adherence); and it is interesting that in discussion they then focus on the data rather than the technology. Our current goal is to exploit the potential for combination of physiological and environmental data to determine if change of habits can be detected and how this correlates with change in health. We are using this functionality to manage the frail elderly within project inCasa and we propose to present preliminary findings.

